# Liver injury after pulsed methylprednisolone therapy in multiple sclerosis patients

**DOI:** 10.1002/brb3.968

**Published:** 2018-05-04

**Authors:** Viviana Nociti, Marco Biolato, Chiara De Fino, Assunta Bianco, Francesco Antonio Losavio, Matteo Lucchini, Giuseppe Marrone, Antonio Grieco, Massimiliano Mirabella

**Affiliations:** ^1^ Multiple Sclerosis Center Neuroscience Area, Neuroscience, Aging, Head and Neck and Orthopaedics Sciences Department Fondazione Policlinico Universitario Gemelli Catholic University of Sacred Heart Rome Italy; ^2^ Fondazione Don Gnocchi‐ONLUS Milan Italy; ^3^ Liver Transplant Medicine Unit Gastroenterological Area, Gastroenterological and Endocrino‐Metabolical Sciences Department Fondazione Policlinico Universitario Gemelli Catholic University of Sacred Heart Rome Italy

**Keywords:** liver injury, multiple sclerosis, relapse, steroids

## Abstract

**Objectives:**

High‐dose pulsed methylprednisolone‐related liver injury cases have been reported in the literature, but a prospective study in patients with multiple sclerosis (MS) has never been performed. The aim of this study was to evaluate the prevalence and severity of liver injury in patients with MS after pulsed methylprednisolone therapy.

**Methods:**

We performed a prospective observational single‐center study on patients with MS treated with i.v. methylprednisolone 1,000 mg/day for 5 days. We tested the liver functionality before and 2 weeks after the treatment. In case of severe liver injury, defined according to “Hy's law,” a comprehensive hepatologic workup was performed.

**Results:**

During a 12‐month observation period, we collected data on 251 cycles of i.v. steroid treatment of 175 patients with MS. After excluding eight cycles presenting a basal alteration of the biochemical liver tests, we observed a prevalence of 8.6% of liver injury in MS patients treated with pulsed methylprednisolone for clinical and neuroradiological relapses. In 2.5% of the patients, the liver injury was severe according to Hy's law; after a comprehensive hepatologic workup, three of them received a diagnosis of drug‐induced liver injury and the other three of autoimmune hepatitis.

**Conclusions:**

Liver injury after pulsed methylprednisolone therapy in patients with MS is not infrequent, and a close monitoring of aminotransferase level before treatment and 2 weeks later seems advisable.

## 1 INTRODUCTION

Multiple sclerosis (MS) is a chronic inflammatory disease of the central nervous system characterized by demyelination and axonal loss (Kamm, Uitdehaag, & Polman, 2014). The clinical course of MS is characterized by relapses and/or disease progression. Relapses are defined as a new or a worsening neurological deficit lasting 24 hr or more in the absence of fever or infections signs (Polman et al., 2010). Most of MS exacerbations are usually followed by a period of repair leading to a clinical remission, and in some cases, to a complete recovery (Berkovich, 2013). However, the residual deficits after a MS relapse may persist contributing to the stepwise progression of disability; therefore, treating MS relapses is important as it may help shortening the disability associated with their course as well as impacting subsequent disease progression (Lublin, Baier, & Cutter, 2003). Corticosteroids are the main pharmacological treatment of MS relapses with a demonstrated effect, eventually supplemented by a plasmapheresis cycle (3–5 treatments) in nonresponders patients (Galea, Ward‐Abel, & Heesen, 2015). A high‐dose short‐term methylprednisolone therapy is the currently recommended first‐line treatment, acting by reducing the inflammation in the brain and the spinal cord, thus increasing the probability of ameliorating the clinical episode speeding up the patient recovery (Citterio et al., 2000; Sellebjerg et al., 2005).

Several different side effects are attributed to corticosteroids, many of which are dose and duration dependent (Fardet, Kassar, Cabane, & Flahault, 2007; Schäcke, Döcke, & Asadullah, 2002). Focusing specifically on a short‐term (pulsed) treatment of high‐dose intravenous methylprednisolone, the adverse events mainly described in the literature include hyperglycemia, tachycardia, flushing, gastrointestinal symptoms, sleep disturbance, psychotic reactions, neutrophilia, and lymphopenia (Acar et al., 2012; Gal, Vedula, & Beck, 2015; Lal et al., 2016; Lienert et al., 2013; Shaygannejad et al., 2013).

High‐dose pulsed methylprednisolone treatment has been also associated with liver injury. By reviewing the literature published in English, we found a total of forty‐seven cases of methylprednisolone‐related liver injury, including patients treated for diseases other than MS (Carrier et al., 2013; D'Agnolo & Drenth, 2013; Das, Graham, & Rose, 2006; Davidov et al., 2016; Dourakis, Sevastianos, & Kaliopi, 2002; Dumortier et al., 2017; Eguchi et al., 2015; Ferraro et al., 2015; Furutama et al., 2011; Gerolami et al., 1997; Grilli, Galati, Petrosillo, Del Nonno, & Baiocchini, 2015; Gutkowski, Chwist, & Hartleb, 2011; Hidalgo de la Cruz et al., 2017; Hofstee, Nanayakkara, & Stehouwer, 2005; Loraschi et al., 2010; Maàmouri et al., 2009; Marinó et al., 2004; Melamud, Lurie, Goldin, Levi, & Esayag, 2014; Moleti et al., 2016; Nanki, Koike, & Miyasaka, 1999; Oliveira, Lopes, Cipriano, & Sofia, 2015; Reuβ, Retzlaff, Vogel, Franke, & Oschmann, 2007; Rivero Fernández et al., 2008; Salvi et al., 2004; Takahashi et al., 2008; Topal et al., 2006; Weissel & Hauff, 2000). The aim of this prospective study was to evaluate the prevalence, severity, and risk factors for liver injury in patients affected by MS treated with pulsed methylprednisolone.

## 2 METHODS

We performed a prospective observational study on adult patients with MS (defined according to the 2010 revisions of McDonald criteria) (Polman et al., 2010) treated with methylprednisolone pulses for clinical and neuroradiological relapses (including disease onset) from April 2014 to April 2015 at the multiple sclerosis center of the “Fondazione Policlinico Universitario Agostino Gemelli,” Catholic University of Sacred Heart, in Rome, Italy. All the patients with MS who experienced a relapse and with no contraindications to methylprednisolone treatment have been enrolled. All patients received i.v. methylprednisolone at the dosage of 1,000 mg/day for 5 days. Biochemical liver function tests, namely aspartate aminotransferase (AST, normal value 7–45 IU/L), alanine aminotransferase (ALT, 7–45 IU/L), total bilirubin (normal value 0.3–1.2 mg/dl), alkaline phosphatase (ALP, normal value 40–129 IU/L), were performed in our laboratory before treatment and 2 weeks after the end of therapy. In case of abnormality, liver function tests were repeated every 2 weeks until normalization.

### 2.1 Definitions

Liver injury was defined as elevation of ALT (with or without abnormalities of the other liver function tests) above the upper normal limit of our local laboratory (45 UI/L). Causality of ALT elevation was assessed through the Naranjo scale (Naranjo et al., 1981) and the World Health Organization (WHO) ‐ Uppsala Monitoring Centre (UMC) causality assessment.

In case of a severe liver injury, defined according to “Hy's law” (hepatocellular injury of any grade associate with bilirubin increase) (Navarro & Senior, 2006), a comprehensive hepatological workup was performed. A careful medical history was collected, including previous exposure to methylprednisolone, concomitant diseases, eating habits, alcohol consumption, eventual consumption of mushrooms, and a detailed history on the use of prescribed and nonprescribed over the counter herbal and other medications or remedies, including time and quantity of substance intake. Performed blood tests included biochemical liver tests, complete blood count, coagulation tests, serum protein electrophoresis, microbiological screening for hepatitis A virus (IgM and IgG antibodies), hepatitis B virus (HBs antigen, HBc and HBs antibodies, HBV‐DNA), hepatitis C virus (HCV‐RNA, anti‐HCV antibodies), hepatitis E virus (IgG antibodies), herpes simplex virus 1 and 2 (IgM and IgG antibodies), varicella zoster virus (IgM and IgG antibodies), Epstein–Barr virus (IgM‐VCA, IgG‐VCA, IgG‐EBNA antibodies), adenovirus (IgA and IgG antibodies), parvovirus B19 (IgM e IgG antibodies), cytomegalovirus (IgM e IgG antibodies), leptospirosis (IgM and IgG antibodies), salmonellosis and brucellosis (Widal–Wright), rickettsioses (Weil–Felix), immunological screening (antinuclear, antismooth muscle, antimitochondrial, antiliver kidney microsomal, antiendomysial, antitransglutaminase antibodies), and metabolic screening (serum ferritin, copper, ceruloplasmin, and alfa‐1‐antitrypsin level). The patients underwent a liver ultrasound examination in order to exclude biliary abnormality, arterial thrombosis or Budd–Chiari syndrome. When clinically appropriate, a liver biopsy was performed and, in selected cases, repeated.

Liver injury was classified as hepatocellular, cholestatic, or mixed according to *R*‐ratio score (*R* = (ALT value/ALT upper normal limit)/(ALP value/ALP upper normal limit); briefly, *R* ratios of >5 define a hepatocellular pattern of liver injury, <2 a cholestatic pattern of liver injury and between 2 and 5 a mixed pattern of liver injury (Chalasani et al., 2014).

The probability of a drug‐induced liver injury (DILI) was assessed by the Roussel UCLAF Causality Assessment Method (RUCAM) that depends on the value of *R*‐ratio and assigns a score to factors such as time to onset, course, risk factors, concomitant drugs, nondrug causes of liver injury, previous information on the hepatotoxicity of the drug and response to rechallenge; briefly, RUCAM indicate that a drug is a possible (Berkovich, 2013; Galea et al., 2015; Lublin et al., 2003), probable (Citterio et al., 2000; Schäcke et al., 2002; Sellebjerg et al., 2005), or highly probable (>8) cause of liver injury (Hayashi & Fontana, 2014).

Diagnosis of autoimmune hepatitis was carried out by Autoimmune Hepatitis Scoring System (IAHG) criteria that assign a score to factors such as clinical features, biochemical liver tests alterations, immunoglobulin, autoantibodies, concomitant autoimmune disease, alternative cause of liver injury, and response to treatment; briefly, IAHG score indicates that an autoimmune hepatitis is probable (Acar et al., 2012; D'Agnolo & Drenth, 2013; Lal et al., 2016; Lienert et al., 2013; Reuβ et al., 2007; Topal et al., 2006) or definite (>17) (Alvarez et al., 1999).

### 2.2 Ethics statement

The local ethic committee approved the study, and all patients signed an informed consent.

### 2.3 Statistical analyses

Independent‐samples Student's *t* test and chi‐squared test were used for group comparisons, as appropriate. Mean ± SD was given for continuous measurements. Frequencies and percentiles were given for categorical data. Binary logistic regression was used to evaluate the risk factors associated with liver injury. Differences were reported as statistically significant if the *p*‐value was less than .05. Statistical analysis was performed using SPSS version 19.0 (SPSS Inc., Chicago, IL).

## 3 RESULTS

### 3.1 Study population

In this study, we included one hundred and seventy five patients treated with pulsed methylprednisolone therapy for a clinical or neuroradiological relapse during 12 months of observation period. The baseline clinical characteristics of the patients are shown in Table 1. The majority of the patients were women (65,1%) with a mean age of 40.8 ± 12.2 years; most of them (73.7%) presented a relapsing–remitting form of MS with a mean duration of disease of 9.7 ± 8.4 years and a mean EDSS score of 2.8 ± 2.1; 61.7% of patients were receiving a disease‐modifying drug (DMD).

**Table 1:** Characteristics of the study population

	All patients (*n* = 175)	Patients with liver injury (*n* = 20^a^)	Patients without liver injury (*n* = 151)	*p*‐value
Age (years)	40.8 ± 12.2	41.5 ± 12.8	40.7 ± 12.1	NS
Female sex (%)	65.1	75	63.6	NS
Smoking habit (%)	14.6	0.0	16.6	.049
Duration of disease (years)	9.7 ± 8.4	6.3 ± 7.4	9.8 ± 8.4	.04
Relapsing‐remitting course (%)	73.7	62.5	75.5	NS
EDSS score prerelapse	2.8 ± 2.1	2.6 ± 2.8	2.9 ± 2.2	NS
EDSS score postrelapse	3.2 ± 1.9	3.1 ± 3.4	3.3 ± 2.0	NS
Disease‐modifying treatment				
Any interferon beta (%)	33.1	29.2	33.8	NS
Glatiramer acetate (%)	12.0	25.0	7.9	.017
Fingolimod (%)	8.0	4.2	7.9	NS
Natalizumab (%)	2.3	0.0	4.0	NS
Other (%)	6.3	0.0	6.0	NS
None (%)	41.7	41.7	41.7	NS

EDSS, expanded disability status scale.

Data presented as number of patients (%) or mean ± SD.

a Four patients presented an alteration of basal alanine aminotransferase before methylprednisolone treatment and were excluded.

One hundred and twenty‐three patients received a single cycle of pulsed methylprednisolone therapy, thirty‐six patients received two cycles, ten patients received three cycles, five patients received four cycles, and one patient received six cycles (for a total of 251 cycles of pulsed methylprednisolone therapy during the observation period). Four patients (for a total of eight cycles of treatment) presented an alteration of basal ALT before methylprednisolone treatment and, after seven of these cycles of therapy, altered ALT levels have been retained; accordingly, we excluded these cases from further analysis. Therefore, our study was conducted on 171 patients and 243 cycles of pulsed methylprednisolone therapy with normal pretreatment ALT level. Two weeks after the methylprednisolone therapy, serum ALT elevation (any grade) was observed on twenty‐one cycles (prevalence 8.6%). In fifteen cases, ALT elevation was mild, with mean ALT value of 65.5 ± 42.5 IU/L; in all cases of mild ALT elevation, spontaneous normalization of ALT was observed in about a month without any treatment. In six patients (prevalence 2.5%), liver injury was defined as severe according to Hy's law. All six cases presented normal biochemical liver tests before the pulsed methylprednisolone therapy. These patients underwent a comprehensive hepatologic workup: three of them received a final diagnosis of drug‐induced liver injury (DILI) and other three a final diagnosis of autoimmune hepatitis (Table 2).

**Table 2:** Characteristics of the patients with severe liver injury after pulsed methylprednisolone therapy

Case	Age	Sex	Time to peak (days)	Max bilirubin (mg/dl)	Max ALT (UI/L)	Max ALP (UI/L)	*R*‐ratio	Presence of autoantibodies	Concomitant medications	Histology	Rechallenge	RUCAM score	Final diagnosis
1	31	F	75	1.5	932	350	18	None	Glatiramer acetate	NP	NP	5	Possible DILI
2	24	F	105	1.3	778	290	17	ASMA	Ibuprofen	Yes	NP	0	Probable autoimmune hepatitis
3	19	F	35	1.46	1,491	91	22	ANA, ASMA	None	Yes	NP	1	Definite autoimmune hepatits
4	35	F	30	10.8	2,956	290	65	None	None	Yes	Yes	8	Probable DILI with immunoallergic response
5	59	F	90	1.87	838	117	19	None	None	Yes	NP	2	Probable autoimmune hepatitis
6	24	M	90	1.5	929	97	21	None	None	Yes	Yes	10	Highly probable DILI

ALT, alanine aminotrasferase; ALP, alkaline phosphatase; *R*, (ALT value/ALT ULN)/(ALP value/ALP ULN), *R* ratios of >5 define a hepatocellular, <2 a cholestatic and between 2 and 5 a mixed pattern of liver injury; ANA, anti‐nuclear antibodies; ASMA, anti‐smooth muscle antibodies; RUCAM, Roussel Uclaf Causality Assessment Method indicating that a drug is a possible (3–5), probable (6–8) or highly probable (>8) cause of liver injury; DILI, drug‐induced liver injury; NP, not performed.

### 3.2 Drug causality assessment in individual cases

Causality assessment for every case of ALT elevation 2 weeks after methylprednisolone therapy (without basal ALT alteration) was reported as probable according to both Naranjo scale and WHO‐UMC causality assessment (Table 3).

**Table 3:** Causality assessment criteria

WHO‐UMC causality criteria	ALT elevation (*n* = 21)	Naranjo scale	ALT elevation (*n* = 21)
Certain	0 (0%)	Definite (≥9)	0 (0%)
Probable	21 (100%)	Probable (5–8)	21 (100%)
Possible	0 (0%)	Possible (1–4)	0 (0%)
Unlikely	0 (0%)	Doubtful (≤0)	0 (0%)

Data presented as number of patients and percentage (%).

WHO, world health organization; UMC, Uppsala monitoring centre; ALT, alanine aminotransferase.

### 3.3 Clinical characteristics of patients with severe liver injury

*Patient 1* was a 31‐year‐old woman, treated with glatiramer acetate, who presented an increase in the AST (49 IU/L) and ALT (110 IU/L) values 2 weeks after the pulsed methylprednisolone therapy. Biochemical liver tests progressively increased, but she remained asymptomatic. Glatiramer acetate was stopped, and ademetionine and ursodeoxycholic acid were administered. Hepatological workup showed a mild steatosis at the liver ultrasound and the presence in the serum of IgM against Citomegalovirus, stably positive on repeated monitoring but associated with the absence of CMV‐DNA by polymerase chain reaction (probably a false‐positive antibody test). In 4 months, the liver function tests returned to normal, and a diagnosis of possible methylprednisolone‐induced liver injury was made.

*Patient 2* was a 24‐year‐old woman, not receiving any DMD for MS, who developed a slowly progressive increase in liver function test after a pulsed methylprednisolone course. She was asymptomatic and reported occasional use of ibuprofen for dysmenorrhea. Autoimmune screening documented a positivity for antismooth muscle antibodies. The liver biopsy showed the presence of interface hepatitis with centrolobular necrosis, consistent with a diagnosis of probable autoimmune hepatitis (IAHG score 13). The patient was treated with ursodeoxycholic acid and started a DMD with glatiramer acetate. Six months later, liver function tests were normal.

*Patient 3* was a 19‐year‐old woman, not taking any DMD for MS, who developed a progressive increase in biochemical liver tests. She was asymptomatic. Immunological tests revealed the presence of antinuclear and antismooth muscle antibodies. Gamma‐globulin were 18.7% of total globulins. A liver biopsy showed a severe centrolobular necrosis and histiocytes activation. Glutathione sodium salt 600 mg i.v. was administered once a week for three times, followed by a therapy with tocopherol and ursodeoxycholic acid for 6 months. Serum ALT levels decreased, but remained three times above the normal values 6 months after methylprednisolone treatment. Liver biopsy was repeated, showing interface hepatitis, centrolobular necrosis, and histiocytes activation, suggestive for autoimmune hepatitis. The patient was treated with azathioprine and budesonide, with a complete normalization of liver function tests 3 months later. A diagnosis of definite autoimmune hepatitis was made (IAHG score 19). She tapered and suspended budesonide and started glatiramer acetate in association with azathioprine.

*Patient 4*, a 35‐year‐old woman not receiving any DMD for MS, presented a progressive increase in biochemical liver tests after pulsed methylprednisolone therapy. She was asymptomatic. The patient had a previous history of liver injury after pulsed steroid therapy on 2009. Liver ultrasound was suggestive for mild steatosis. Autoantibodies were negative. Liver biopsy showed interface hepatitis, submassive necrosis, severe fibrosis; a final diagnosis was made of a probable drug‐related hepatitis with immunoallergic response.

*Patient 5* was a 59‐year‐old woman, not receiving any DMD for MS, who presented ALT abnormality (48 IU/L) 2 weeks after pulsed methylprednisolone therapy, reaching a peak of 838 IU/L 3 months later, while she remained asymptomatic. Hepatological workup was unremarkable except for a mild steatosis on liver ultrasound. Liver biopsy showed a severe hepatitis with necrosis and histiocytes activation. She was treated with ademetionine. Serum ALT levels decreased although remaining four times above the normal values 6 months after methylprednisolone treatment. Liver biopsy was then repeated, showing mild hepatitis, fibrosis, and histiocytes activation without plasma cells infiltration. The patient was treated with azathioprine and budesonide with complete normalization of liver function test 6 months later. A final diagnosis of probable autoimmune hepatitis was made (IAHG score 17). She tapered and suspended budesonide and continued the azathioprine monotherapy.

*Patient 6* was a 24‐year‐old man who presented a progressive increase in biochemical liver tests after pulsed methylprednisolone therapy. The patient had already a history of liver injury after pulsed steroid therapy on 2013 (ALT peak 2,850 IU/L) and again on 2014 (ALT peak 1,947 IU/L). Hepatologic workup showed ferritin level of 1,053 ng/ml with iron saturation of 55%, but abdominal magnetic resonance imaging showed normal liver iron content. Liver biopsy showed centrolobular necrosis, mild hepatitis and fibrosis and histiocytes activation. He was treated with ademetionin and ursodeoxycholic acid. Six months later, liver function was normal. A diagnosis of highly probable drug‐induced liver injury was made. The patient started DMD with fingolimod without subsequent elevation of the biochemical liver tests.

### 3.4 Univariate analysis and binary logistic regression analysis for factors associated with DILI outcomes

Univariate analysis (Table 1) shows that the population of patients with liver injury (*n* = 20) and the population of patients without liver injury (*n* = 151) were similar for most of their clinical features, except for the disease duration (shorter in the liver injury group), a smoking habit (present only in patients without liver injury), and a concomitant treatment with glatiramer acetate (more frequently in the liver injury group).

To further investigate the risk factors associated with liver injury, a multivariate binary logistic regression analysis was performed, including age, sex, smoking habit, disease duration, clinical form of MS, and disease‐modifying treatment. No positive or negative association was found between these variables and risk of liver injury.

## 4 DISCUSSION

Our study showed a prevalence of 8.6% of liver injury in patients affected by MS and treated with pulsed methylprednisolone for clinical and neuroradiological relapses. In 2.5% of patients, the liver injury was severe (according to Hy's law) and attributable either to a drug‐induced liver injury or to the onset of an autoimmune hepatitis.

To the best of our knowledge, this is the first prospective observation study on methylprednisolone hepatotoxicity in a population of patients with MS, so it is not possible to make direct comparisons with previous work in the literature reporting hepatotoxicity cases as adverse event of methylprednisolone administration in MS (Hidalgo de la Cruz et al., 2017). Eguci and coworkers described a prevalence of 45% of ALT increase and of 4% of severe liver dysfunction in a cohort of 175 Japanese patients treated with methylprednisolone pulse therapy for Graves’ ophthalmopathy, but they included many patients with chronic HBV or HCV hepatitis (Eguchi et al., 2015).

We analyzed separately the population of patients with and without liver injury in order to find relevant risk factors for liver injury. Both populations were in fact quite similar for most of the clinical features, but we were not able to identify any risk or protective factor also because of the limited number of events observed.

Methylprednisolone's labelling information mentions transient increases in serum transaminases and hepatomegaly, but not clinically relevant hepatotoxicity; however, the literature review of high‐dose pulsed methylprednisolone (for any therapeutic indication) identified forty‐seven cases of liver injury, including one death (Carrier et al., 2013; Caster, Conforti, Viola, & Edwards, 2014; D'Agnolo & Drenth, 2013; Das et al., 2006; Davidov et al., 2016; Dourakis et al., 2002; Dumortier et al., 2017; Eguchi et al., 2015; Ferraro et al., 2015; Furutama et al., 2011; Gerolami et al., 1997; Grilli et al., 2015; Gutkowski et al., 2011; Hidalgo de la Cruz et al., 2017; Hofstee et al., 2005; Loraschi et al., 2010; Maàmouri et al., 2009; Marinó et al., 2004; Melamud et al., 2014; Moleti et al., 2016; Nanki et al., 1999; Oliveira et al., 2015; Reuβ et al., 2007; Rivero Fernández et al., 2008; Salvi et al., 2004; Takahashi et al., 2008; Topal et al., 2006; Weissel & Hauff, 2000).

In case of severe methylprednisolone‐related liver injury, a careful hepatic workup is warranted in order to accurately attribute the responsibility of the toxic effect to the drug rather than to the an ex novo onset of autoimmune hepatitis (Carrier et al., 2013; Maàmouri et al., 2009; Reuβ et al., 2007; Salvi et al., 2004; Takahashi et al., 2008). In our cohort, three patients received a final diagnosis of probable or definite autoimmune hepatitis (patients 2, 3, and 5) and their baseline clinical characteristics were almost indistinguishable from those of patients who received a final diagnosis of DILI.. The diagnostic challenge is made even more difficult by the fact that there is a continuous spectrum crossing the diagnosis of hepatoxicity with immunoallergic mechanism with autoimmune hepatitis due to immune rebound phenomenon (De Boer et al., 2017). The distinction between these two pathological entities has an important therapeutic relevance for the neurologist. In case of methylprednisolone‐induced liver injury, the neurologist has to take this into account to set the goal of reducing the clinical disease activity as much as possible. In fact, as the typically relapsing course of MS carries the need to repeat pulsed methylprednisolone treatment several times, in patient with high disease activity an early treatment with a second‐line DMD such as natalizumab or fingolimod should be considered to more effectively reduce disease activity. The use of alemtuzumab in these patients may be limited by the need to coadminister methylprednisolone during the infusion cycle. In case of severe relapse, a rescue therapy with plasma exchange should be considered in patients with methylprednisolone‐induced liver injury.

In case of autoimmune hepatitis, instead, a concomitant treatment with azathioprine represents an important therapeutic limitation to the use of all second‐line DMD. In addition, interferon beta is contraindicated in autoimmune hepatitis because of the possible worsening of liver disease activity. Treatment with glatiramer acetate is an option. There are no available safety data on the association of azathioprine with dimethyl‐fumarate, neither on the use of teriflunomide in autoimmune hepatitis instead of azathioprine.

Our data highlight the importance of a close follow‐up of the liver function tests following pulsed methylprednisolone treatment. According to our experience, we suggest to test transaminases 2 weeks after pulsed methylprednisolone treatment in all patients, and particularly, before the beginning of a new DMD therapy, in order to avoid a misdiagnosis of DMD‐induced liver injury. In case of abnormality, the start of the DMD therapy should be postponed and biochemical liver tests have to be repeated until normalization, while it is advisable to refer the patient to a hepatologist in case of bilirubin elevation.

In conclusion, our study confirms that liver injury after pulsed methylprednisolone therapy in patients with MS is not infrequent. A complete baseline assessment of the liver function and a close monitoring after each cycle of high‐doses methylprednisolone is recommended. In our experience, 2 weeks later seems to be the right interval for such monitoring.

## CONFLICT OF INTEREST

None to be declared.

## References

[brb3968-bib-0001] Acar, M. , Gedizlioglu, M. , Koskderelioglu, A. , Ozturk, F. , Kilinc, S. , & Talay, N. (2012). Effect of high‐dose intravenous methyl‐prednisolone treatment on intraocular pressure in multiple sclerosis patients with relapse. European Neurology, 68, 20–22. https://doi.org/10.1159/000337615 22677889 10.1159/000337615

[brb3968-bib-0002] Alvarez, F. , Berg, P. A. , Bianchi, F. B. , Bianchi, L. , Burroughs, A. K. , Cancado, E. L. , … MacSween, R. N. (1999). International Autoimmune Hepatitis Group Report: Review of criteria for diagnosis of autoimmune hepatitis. Journal of Hepatology, 31, 929–938. https://doi.org/10.1016/S0168-8278(99)80297-9 10580593 10.1016/s0168-8278(99)80297-9

[brb3968-bib-0003] Berkovich, R. (2013). Treatment of acute relapses in multiple sclerosis. Neurotherapeutics: The Journal of the American Society for Experimental NeuroTherapeutics, 10, 97–105. https://doi.org/10.1007/s13311-012-0160-7 23229226 10.1007/s13311-012-0160-7 PMC3557364

[brb3968-bib-0004] Carrier, P. , Godet, B. , Crepin, S. , Magy, L. , Debette‐Gratien, M. , Pillegand, B. , … Loustaud‐Ratti, V. (2013). Acute liver toxicity due to methylprednisolone: Consider this diagnosis in the context of autoimmunity. Clinics and Research in Hepatology and Gastroenterology, 37, 100–104. https://doi.org/10.1016/j.clinre.2012.10.015 23318289 10.1016/j.clinre.2012.10.015

[brb3968-bib-0005] Caster, O. , Conforti, A. , Viola, E. , & Edwards, I. R. (2014). Methylprednisolone‐induced hepatotoxicity: Experiences from global adverse drug reaction surveillance. European Journal of Clinical Pharmacology, 70, 501–503. https://doi.org/10.1007/s00228-013-1632-3 24384565 10.1007/s00228-013-1632-3

[brb3968-bib-0006] Chalasani, N. P. , Hayashi, P. H. , Bonkovsky, H. L. , Navarro, V. J. , Lee, W. M. , Fontana, R. J. ; Practice Parameters Committee of the American College of Gastroenterology (2014). ACG Clinical Guideline: The diagnosis and management of idiosyncratic drug‐induced liver injury. American Journal of Gastroenterology, 109, 950–966. https://doi.org/10.1038/ajg.2014.131 24935270 10.1038/ajg.2014.131

[brb3968-bib-0007] Citterio, A. , La Mantia, L. , Ciucci, G. , Brusaferri, F. , Sibley, W. A. , Midgard, R. , & Candelise, L. (2000). Corticosteroids or ACTH for acute exacerbations in multiple sclerosis. Cochrane Database of Systematic Reviews, (4), CD001331 https://doi.org/10.1002/14651858.CD001331 11034713 10.1002/14651858.CD001331 PMC11391333

[brb3968-bib-0008] D'Agnolo, H. M. , & Drenth, J. P. (2013). High‐dose methylprednisolone‐induced hepatitis in a patient with multiple sclerosis: A case report and brief review of literature. Netherlands Journal of Medicine, 71, 199–202. 23723114

[brb3968-bib-0009] Das, D. , Graham, I. , & Rose, J. (2006). Recurrent acute hepatitis in patient receiving pulsed methylprednisolone for multiple sclerosis. Indian Journal of Gastroenterology, 25, 314–316. 17264438

[brb3968-bib-0010] Davidov, Y. , Har‐Noy, O. , Pappo, O. , Achiron, A. , Dolev, M. , & Ben‐Ari, Z. (2016). Methylprednisolone‐induced liver injury: Case report and literature review. Journal of Digestive Diseases, 17, 55–62. https://doi.org/10.1111/1751-2980.12306 26676833 10.1111/1751-2980.12306

[brb3968-bib-0011] De Boer, Y. S. , Kosinski, A. S. , Urban, T. J. , Zhao, Z. , Long, N. , Chalasani, N. , … Hoofnagle, J. H. (2017). Drug‐Induced liver injury network features of autoimmune hepatitis in patients with drug‐induced liver injury. Clinical Gastroenterology and Hepatology, 15, 103–112. https://doi.org/10.1016/j.cgh.2016.05.043 27311619 10.1016/j.cgh.2016.05.043 PMC5370577

[brb3968-bib-0012] Dourakis, S. P. , Sevastianos, V. A. , & Kaliopi, P. (2002). Acute severe steatohepatitis related to prednisolone therapy. American Journal of Gastroenterology, 97, 1074–1075. https://doi.org/10.1111/j.1572-0241.2002.05644.x 12003403 10.1111/j.1572-0241.2002.05644.x

[brb3968-bib-0013] Dumortier, J. , Cottin, J. , Lavie, C. , Guillaud, O. , Hervieu, V. , Chambon‐Augoyard, C. , … Vial, T. (2017). Methylprednisolone liver toxicity: A new case and a French regional pharmacovigilance survey. Clinics and Research in Hepatology and Gastroenterology, 41, 497–501. https://doi.org/10.1016/j.clinre.2017.03.008 28438569 10.1016/j.clinre.2017.03.008

[brb3968-bib-0014] Eguchi, H. , Tani, J. , Hirao, S. , Tsuruta, M. , Tokubuchi, I. , Yamada, K. , … Hiromatsu, Y. (2015). Liver dysfunction associated with intravenous methylprednisolone pulse therapy in patients with Graves’ orbitopathy. International Journal of Endocrinology, 2015, 835979. 26221141 10.1155/2015/835979 PMC4499413

[brb3968-bib-0015] Fardet, L. , Kassar, A. , Cabane, J. , & Flahault, A. (2007). Corticosteroid‐induced adverse events in adults: Frequency, screening and prevention. Drug Safety, 30, 861–881. https://doi.org/10.2165/00002018-200730100-00005 17867724 10.2165/00002018-200730100-00005

[brb3968-bib-0016] Ferraro, D. , Mirante, V. G. , Losi, L. , Villa, E. , Simone, A. M. , Vitetta, F. , … Sola, P. (2015). Methylprednisolone‐induced toxic hepatitis after intravenous pulsed therapy for multiple sclerosis relapses. Neurologist, 19, 153–154. https://doi.org/10.1097/NRL.0000000000000029 26075468 10.1097/NRL.0000000000000029

[brb3968-bib-0017] Furutama, D. , Kimura, F. , Shinoda, K. , Maeda, T. , Tanaka, T. , & Ohsawa, N. (2011). Recurrent high‐dose intravenous methylprednisolone succinate pulse therapy‐induced hepatopathy in a patient with multiple sclerosis. Medical Principles and Practice, 20, 291–293. https://doi.org/10.1159/000323835 21455003 10.1159/000323835

[brb3968-bib-0018] Gal, R. L. , Vedula, S. S. , & Beck, R. (2015). Corticosteroids for treating optic neuritis. The Cochrane Database of Systematic Reviews, 8, CD001430. 10.1002/14651858.CD001430.pub4 PMC4730547 26273799

[brb3968-bib-0019] Galea, I. , Ward‐Abel, N. , & Heesen, C. (2015). Relapse in multiple sclerosis. BMJ, 350, h1765 https://doi.org/10.1136/bmj.h1765 25872511 10.1136/bmj.h1765

[brb3968-bib-0020] Gerolami, R. , Mambrini, P. , Barthet, M. , Jean‐Pastor, M. J. , Salducci, J. , & Grimaud, J. C. (1997). Acute hepatitis caused by Solupred in a patient with Crohn disease. Gastroenterologie Clinique et Biologique, 21, 236–237. 9161506

[brb3968-bib-0021] Grilli, E. , Galati, V. , Petrosillo, N. , Del Nonno, F. , & Baiocchini, A. (2015). Incomplete septal cirrhosis after high‐dose methylprednisolone therapy and regression of liver injury. Liver International: Official Journal of the International Association for the Study of the Liver, 35, 674–676. https://doi.org/10.1111/liv.12607 24906135 10.1111/liv.12607

[brb3968-bib-0022] Gutkowski, K. , Chwist, A. , & Hartleb, M. (2011). Liver injury induced by high‐dose methylprednisolone therapy: A case report and brief review of the literature. Hepatitis Monthly, 11, 656–661. https://doi.org/10.5812/kowsar.1735143X 22140391 10.5812/kowsar.1735143X.713 PMC3227489

[brb3968-bib-0023] Hayashi, P. H. , & Fontana, R. J. (2014). Clinical features, diagnosis, and natural history of drug‐induced liver injury. Seminars in Liver Disease, 34, 134–144. 24879979 10.1055/s-0034-1375955

[brb3968-bib-0024] Hidalgo de la Cruz, M. , Miranda Acuña, J. A. , Lozano Ros, A. , Vega Catalina, M. , Salinero Paniagua, E. , Clemente Ricote, G. , … Martínez Ginés, M. L. (2017). Hepatotoxicity after high‐dose intravenous methylprednisolone in multiple sclerosis patients. Clinical Case Reports, 5, 1210–1212. https://doi.org/10.1002/ccr3.1033 28781825 10.1002/ccr3.1033 PMC5538224

[brb3968-bib-0025] Hofstee, H. M. , Nanayakkara, P. W. , & Stehouwer, C. D. (2005). Acute hepatitis related to prednisolone. European Journal of Internal Medicine, 16, 209–210. https://doi.org/10.1016/j.ejim.2004.10.018 15967341 10.1016/j.ejim.2004.10.018

[brb3968-bib-0026] Kamm, C. P. , Uitdehaag, B. M. , & Polman, C. H. (2014). Multiple sclerosis: Current knowledge and future outlook. European Neurology, 72, 132–141. https://doi.org/10.1159/000360528 25095894 10.1159/000360528

[brb3968-bib-0027] Lal, R. , Bell, S. , Challenger, R. , Hammock, V. , Nyberg, M. , Decker, D. , … Young, D. (2016). Pharmacodynamics and tolerability of repository corticotropin injection in healthy human subjects: A comparison with intravenous methylprednisolone. Journal of Clinical Pharmacology, 56, 195–202. https://doi.org/10.1002/jcph.582 26120075 10.1002/jcph.582 PMC5049675

[brb3968-bib-0028] Lienert, C. , Schawalder, G. , Findling, O. , Kamm, C. P. , Humpert, S. , Mugglin, A. , … Mattle, H. P. (2013). Tolerance of intravenous methylprednisolone for relapse treatment in demyelinating CNS disease. Swiss Medical Weekly, 143, w13783. 23740464 10.4414/smw.2013.13783

[brb3968-bib-0029] Loraschi, A. , Banfi, P. , Mauri, M. , Sessa, F. , Bono, G. , & Cosentino, M. (2010). Hepatotoxicity after high‐dose methylprednisolone for demyelinating disease. Clinical Neuropharmacology, 33, 52–54. https://doi.org/10.1097/WNF.0b013e3181bbf3a7 19935411 10.1097/WNF.0b013e3181bbf3a7

[brb3968-bib-0030] Lublin, F. D. , Baier, M. , & Cutter, G. (2003). Effect of relapses on development of residual deficit in multiple sclerosis. Neurology, 61, 1528–1532. https://doi.org/10.1212/01.WNL.0000096175.39831.21 14663037 10.1212/01.wnl.0000096175.39831.21

[brb3968-bib-0031] Maàmouri, N. , Ketari, S. , Ben Hariz, F. , Belkahla, N. , Romdhane, S. B. , Chouaib, S. , … Mami, N. B. (2009). Bolus de méthylprednisolone révélant une hépatite auto‐immune chez une patiente atteinte de sclérose en plaques. Journal Africain D'Hépato‐Gastroentérologie, 3, 195–197. https://doi.org/10.1007/s12157-009-0116-7

[brb3968-bib-0032] Marinó, M. , Morabito, E. , Brunetto, M. R. , Bartalena, L. , Pinchera, A. , & Marocci, C. (2004). Acute and severe liver damage associated with intravenous glucocorticoid pulse therapy in patients with Graves’ ophthalmopathy. Thyroid, 14, 403–436. https://doi.org/10.1089/105072504774193276 15186621 10.1089/105072504774193276

[brb3968-bib-0033] Melamud, B. , Lurie, Y. , Goldin, E. , Levi, I. , & Esayag, Y. (2014). Methylprednisolone‐induced liver injury: A diagnostic challenge. The Israel Medical Association Journal, 16, 180–181. 24761710

[brb3968-bib-0034] Moleti, M. , Giuffrida, G. , Sturniolo, G. , Squadrito, G. , Campennì, A. , Morelli, S. , … Marinò, M. (2016). Acute liver damage following intravenous glucocorticoid treatment for Graves’ ophthalmopathy. Endocrine, 54, 259–268. https://doi.org/10.1007/s12020-016-0928-3 27003434 10.1007/s12020-016-0928-3

[brb3968-bib-0035] Nanki, T. , Koike, R. , & Miyasaka, N. (1999). Subacute severe steatohepatitis during prednisolone therapy for systemic lupus erythematosis. American Journal of Gastroenterology, 94, 3379 https://doi.org/10.1111/j.1572-0241.1999.03379.x 10.1111/j.1572-0241.1999.03379.x 10566758

[brb3968-bib-0036] Naranjo, C. A. , Busto, U. , Sellers, E. M. , Sandor, P. , Ruiz, I. , Roberts, E. A. , … Greenblatt, D. J. (1981). A method for estimating the probability of adverse drug reactions. Clinical Pharmacology and Therapeutics, 30, 239–245. https://doi.org/10.1038/clpt.1981.154 7249508 10.1038/clpt.1981.154

[brb3968-bib-0037] Navarro, V. J. , & Senior, J. R. (2006). Drug‐related hepatotoxicity. New England Journal of Medicine, 354, 731–739. https://doi.org/10.1056/NEJMra052270 16481640 10.1056/NEJMra052270

[brb3968-bib-0038] Oliveira, A. T. , Lopes, S. , Cipriano, M. A. , & Sofia, C. (2015). Induced liver injury after high‐dose methylprednisolone in a patient with multiple sclerosis. BMJ Case Reports, 21, 2015. 10.1136/bcr-2015-210722 PMC4513529 26199298

[brb3968-bib-0039] Polman, C. H. , Reingold, S. C. , Banwell, B. , Clanet, M. , Cohen, J. A. , Filippi, M. , … Wolinsky, J. S. (2010). Diagnostic criteria for multiple sclerosis: 2010 revisions to the McDonald criteria. Annals of Neurology, 69, 292–302. 10.1002/ana.22366 PMC3084507 21387374

[brb3968-bib-0040] Reuβ, R. , Retzlaff, K. , Vogel, S. , Franke, F. E. , & Oschmann, P. (2007). Autoimmune hepatitis after high‐dose intravenous methylprednisolone pulse in RR‐MS. Central European Journal of Medicine, 2, 356–359.

[brb3968-bib-0041] Rivero Fernández, M. , Riesco, J. M. , Moreira, V. F. , Moreno, A. , López San Román, A. , Arranz, G. , & Ruiz Del Arbol, L. (2008). Recurrent acute liver toxicity from intravenous methylprednisolone. Revista Espanola de Enfermedades Digestivas, 100, 720–723. 19159178 10.4321/s1130-01082008001100010

[brb3968-bib-0042] Salvi, M. , Vannucchi, G. , Sbrozzi, F. , Del Castello, A. B. , Carnevali, A. , Fargion, S. , & Beck‐Peccoz, P. (2004). Onset of autoimmune hepatitis during intravenous steroid therapy for thyroid‐associated ophthalmopathy in a patient with Hashimoto's thyroiditis: Case report. Thyroid, 14, 631–634. https://doi.org/10.1089/1050725041692927 15320978 10.1089/1050725041692927

[brb3968-bib-0043] Schäcke, H. , Döcke, W. D. , & Asadullah, K. (2002). Mechanisms involved in the side effects of glucocorticoids. Pharmacology & Therapeutics, 96, 23–43. https://doi.org/10.1016/S0163-7258(02)00297-8 12441176 10.1016/s0163-7258(02)00297-8

[brb3968-bib-0044] Sellebjerg, F. , Barnes, D. , Filippini, G. , Midgard, R. , Montalban, X. , Rieckmann, P. , … Sørensen, P. S. (2005). EFNS guideline on treatment of multiple sclerosis relapses: Report of an EFNS task force on treatment of multiple sclerosis relapses. European Journal of Neurology, 12, 939–946. https://doi.org/10.1111/j.1468-1331.2005.01352.x 16324087 10.1111/j.1468-1331.2005.01352.x

[brb3968-bib-0045] Shaygannejad, V. , Ashtari, F. , Alinaghian, M. , Norouzi, R. , Salari, M. , & Fatehi, F. (2013). Short‐term safety of pulse steroid therapy in multiple sclerosis relapses. Clinical Neuropharmacology, 36, 1–3. https://doi.org/10.1097/WNF.0b013e3182764f91 23334067 10.1097/WNF.0b013e3182764f91

[brb3968-bib-0046] Takahashi, A. , Kanno, Y. , Takahashi, Y. , Sakamoto, N. , Monoe, K. , Saito, H. , … Ohira, H. (2008). Development of autoimmune hepatitis type 1 after pulsed methylprednisolone therapy for multiple sclerosis: A case report. World Journal of Gastroenterology, 14, 5474–5477. https://doi.org/10.3748/wjg.14.5474 18803363 10.3748/wjg.14.5474 PMC2744174

[brb3968-bib-0047] Topal, F. , Özaslan, E. , Akbulut, S. , Küçükazman, M. , Yüksel, O. , & Altıparmak, E. (2006). Methylprednisolone‐induced toxic hepatitis. Annals of Pharmacotherapy, 40, 1868–1871. https://doi.org/10.1345/aph.1H171 16926305 10.1345/aph.1H171

[brb3968-bib-0048] Uppsala Monitoring Centre . The use of the WHO‐UMC system for standardized case causality assessment. Retrieved from http://www.who.int/medicines/areas/quality_safety/safety_efficacy/WHOcausality_assessment.pdf

[brb3968-bib-0049] Weissel, M. , & Hauff, W. (2000). Fatal liver failure after high‐dose glucocorticoid pulse therapy in a patient with severe thyroid eye disease. Thyroid, 10, 521. 10907999 10.1089/thy.2000.10.521

